# A corrected formulation for marginal inference derived from two-part mixed models for longitudinal semi-continuous data

**DOI:** 10.1177/0962280213509798

**Published:** 2013-11-06

**Authors:** Brian DM Tom, Li Su, Vernon T Farewell

**Affiliations:** Medical Research Council Biostatistics Unit, Institute of Public Health, Cambridge, UK

**Keywords:** bridge distribution, excess zeros, longitudinal data, random effects

## Abstract

For semi-continuous data which are a mixture of true zeros and continuously distributed positive values, the use of two-part mixed models provides a convenient modelling framework. However, deriving population-averaged (marginal) effects from such models is not always straightforward. Su *et al.* presented a model that provided convenient estimation of marginal effects for the logistic component of the two-part model but the specification of marginal effects for the continuous part of the model presented in that paper was based on an incorrect formulation. We present a corrected formulation and additionally explore the use of the two-part model for inferences on the overall marginal mean, which may be of more practical relevance in our application and more generally.

## 1 Introduction

In Su *et al.*,^1^ we described a two-part marginal model for longitudinal semi-continuous data that are a mixture of true zeros and continuously distributed positive values. Our likelihood-based model had an underlying two-part mixed model, where, in the random effects logistic regression for the first part (i.e. the binary part), the random intercept was assumed to follow the bridge distribution of Wang and Louis.^2^ A zero-mean normal random intercept was included into the linear mixed modelling structure of the second part (i.e. the continuous part).

Our primary focus was to ensure that the regression parameters in the binary part of the two-part marginal model were interpretable after integration over the random effects distribution. Marginal covariate effects on the expected value of the response for the population of observed non-zero responses may, however, also be of interest. These arise directly from the approaches in Moulton *et al*.,^3^ Lu *et al.*,^4^ Hall and Zhang^5^ and Yang and Simpson^6^ and involve well-defined integrations (see refs^7,8^).

However, when discussing the continuous part of our model, we assumed, as did Tooze *et al.*,^9^ that integrating out the random effects was straightforward, and that the form of the relationship between covariates and the marginal mean of the response, given that it is positive, was the same as the conditional mean given a positive response and random effects. Unfortunately, this is not the case. This paper rectifies this error and explores the use of the proposed model when the target of inference is the overall marginal mean, which may be of most practical relevance.

## 2 Marginal inference from two-part models

### 2.1 Model

Our two-part marginal model^1^ is based on the original two-part mixed modelling framework introduced in Olsen and Schafer^10^ and Tooze *et al*.^9^ and the random effects specifications in Lin *et al*.^11^

Briefly, let *Y_ij_* be a semi-continuous variable for the *i*th (i=1,…,N) subject at time *t_ij_* ($j=1,\ldots ,n_{i}$). Let $X_{\mathrm{ij}}$ and $X_{\mathrm{ij}}^{*}$ be the covariate vectors (possibly overlapping) associated with the *i*th subject at time *t_ij_* in the two parts of the two-part mixed model. Let *B_i_* and *V_i_* be correlated subject-level random intercepts, which are independent of the covariates. Define also $\Omega_{\mathrm{ij}}=\{B_{i},V_{i},X_{\mathrm{ij}},X_{\mathrm{ij}}^{*}\}$ and $\Xi_{\mathrm{ij}}=\{X_{\mathrm{ij}},X_{\mathrm{ij}}^{*}\}$.

*Y_ij_* can be represented by two variables, the occurrence variable $Z_{\mathrm{ij}}=I(Y_{\mathrm{ij}}>0)$ and the intensity variable $g(Y_{\mathrm{ij}})$ given that $Y_{\mathrm{ij}}>0$, where g(·) is a (monotonic) transformation making $Y_{\mathrm{ij}}|Y_{\mathrm{ij}}>0$ normally distributed with a subject–time-specific mean.

The distribution of *Y_ij_* is formulated by assuming, firstly, that *Z_ij_* is specified by a random effects logistic regression with $\text{logit}\{\text{Pr}(Z_{\mathrm{ij}}=1|\Omega_{\mathrm{ij}})\}=X_{\mathrm{ij}}\overset{\sim }{\theta }+B_{i}$, where $X_{\mathrm{ij}}$ is a 1×q covariate vector, $\overset{\sim }{\theta }$ is a q×1 regression coefficient vector and *B_i_* is the subject-level random intercept in this first part (i.e. the binary part) assumed to follow the (symmetric) mean zero bridge distribution of Wang and Louis^2^ with unknown parameter ϕ (0<ϕ<1). Next, the intensity variable $g(Y_{\mathrm{ij}})$ given $Y_{\mathrm{ij}}>0$ is assumed to have the linear mixed modelling structure described by $g(Y_{\mathrm{ij}})|\Omega_{\mathrm{ij}},Y_{\mathrm{ij}}>0=X_{\mathrm{ij}}^{*}\beta +V_{i}+\varepsilon_{\mathrm{ij}}$, where $X_{\mathrm{ij}}^{*}$ is a 1×p covariate vector, β is a p×1 regression coefficient vector and *V_i_* is the subject-level random intercept for the second part (i.e. the continuous part) assumed $N(0,\sigma_{v}^{2})$. The error term $\varepsilon_{\mathrm{ij}}$ is assumed to be $N(0,\sigma_{e}^{2})$ and independent of the random effects. The random effects, *B_i_* and *V_i_* are assumed to be correlated with their joint distribution specified through a Gaussian copula transformation model, where the correlation of the underlying Gaussian random variables is *ρ* (see Supplementary Material). The covariate vectors $X_{\mathrm{ij}},X_{\mathrm{ij}}^{*}$ may coincide, but this is not required.

### 2.2 Marginal covariate effects

The main benefit of the bridge density, stressed in Su *et al*.,^1^ is that after integration over the random intercepts, $\left(B_{i},V_{i}\right)$, the marginal probability $\text{Pr}(Z_{\mathrm{ij}}=1|\Xi_{\mathrm{ij}})$ relates to the linear predictors through the *same* logit link function as for the corresponding conditional probability, $\text{Pr}(Z_{\mathrm{ij}}=1|\Omega_{\mathrm{ij}})$. Furthermore, if we specify the marginal regression structure of the binary part as $\text{logit}\{\text{Pr}(Z_{\mathrm{ij}}=1|\Xi_{\mathrm{ij}})\}=X_{\mathrm{ij}}\theta$, then the marginal covariate effects θ are proportional to the subject-specific conditional covariate effects $\overset{\sim }{\theta }$, with $\theta =\phi \overset{\sim }{\theta }$.

However, although in Su *et al*.^1^ we claimed that the marginal mean of $g(Y_{\mathrm{ij}})|\Xi_{\mathrm{ij}},Y_{\mathrm{ij}}>0$, found after integrating $g(Y_{\mathrm{ij}})|\Omega_{\mathrm{ij}},Y_{\mathrm{ij}}>0$ over $\left(B_{i},V_{i}\right)$, is $X_{\mathrm{ij}}^{*}\beta$, this is not the case generally. The correct form of the marginal mean of $g(Y_{\mathrm{ij}})|\Xi_{\mathrm{ij}},Y_{\mathrm{ij}}>0$ is

(1) $$E\{g(Y_{\mathrm{ij}})|\Xi_{\mathrm{ij}},Y_{\mathrm{ij}}>0\}=X_{\mathrm{ij}}^{*}\beta +E(V_{i}|\Xi_{\mathrm{ij}},Y_{\mathrm{ij}}>0)$$

which will be dependent on the impact of covariates, $X_{\mathrm{ij}}$, on the marginal and conditional probabilities of occurrence (see Supplementary Material).

As the integral given by $E(V_{i}|\Xi_{\mathrm{ij}},Y_{\mathrm{ij}}>0)$ has no closed-form solution, an exact analytical expression for (1) is not available. However, bounds on (1) are available. Specifically, we can show, after some algebraic manipulations (see Supplementary Material), that for ρ≥0

$$X_{\mathrm{ij}}^{*}\beta \leq E(g(Y_{\mathrm{ij}})|\Xi_{\mathrm{ij}},Y_{\mathrm{ij}}>0)\leq X_{\mathrm{ij}}^{*}\beta +\frac{\sigma_{v}\rho }{\sqrt{2\pi }}(1+e-X_{\mathrm{ij}}\theta )$$

and for ρ≤0

$$X_{\mathrm{ij}}^{*}\beta \geq E(g(Y_{\mathrm{ij}})|\Xi_{\mathrm{ij}},Y_{\mathrm{ij}}>0)\geq X_{\mathrm{ij}}^{*}\beta +\frac{\sigma_{v}\rho }{\sqrt{2\pi }}(1+e-X_{\mathrm{ij}}\theta )$$

Although an exact analytical expression is not available, numerically solving (1) at the maximum-likelihood estimates is straightforward as only a single integral is involved. This integral can be evaluated using adaptive Gaussian quadrature techniques. The estimation of the parameters $\theta ,\beta ,\sigma_{b}^{2},\sigma_{v}^{2},\sigma_{e}^{2}$ and *ρ* is based on maximizing the likelihood presented in Su *et al*.^1^

### 2.3 Interpretation of the marginal effects in the continuous part

As noted earlier and in Su *et al.*,^1^ the interpretation of θ is straightforward as these parameters are simply (population-averaged) log-odds ratios. In contrast, assessment of the impact of a covariate on the marginal mean (given being positive), $E(Y_{\mathrm{ij}}|\Xi_{\mathrm{ij}},Y_{\mathrm{ij}}>0)$, depends on whether or not that covariate is also involved in the binary part of the two-part model. If the covariate is not included in the binary part or *B_i_* and *V_i_* are uncorrelated (i.e. ρ=0), then the interpretation of its effect on $E(Y_{\mathrm{ij}}|\Xi_{\mathrm{ij}},Y_{\mathrm{ij}}>0)$ can be quantified through just the appropriate element of β. However when *B_i_* and *V_i_* are correlated and, in addition, the covariate of interest is in both regression components of the model, then a simple interpretation is not readily obtainable because of the non-linearity of (1) in this covariate.

In such a case, the impact of a covariate could be assessed through plotting the relationship between this covariate and $E(Y_{\mathrm{ij}}|\Xi_{\mathrm{ij}},Y_{\mathrm{ij}}>0)$, with other covariates held fixed, or alternatively by describing the local changes (i.e. through the derivative or the difference) in $E(Y_{\mathrm{ij}}|\Xi_{\mathrm{ij}},Y_{\mathrm{ij}}>0)$ with respect to the covariate.^7^ However, the clinical relevance of $E(Y_{\mathrm{ij}}|\Xi_{\mathrm{ij}},Y_{\mathrm{ij}}>0)$ has been questioned, as discussed by Albert^12^ in light of work by Lu *et al.*^4^ and Williamson *et al.*^13^ On the other hand, the overall marginal mean of *Y_ij_* as the target of inference is more easily justified clinically. The calculation of this overall mean is addressed in Section 2.4 and Section 2.5 illustrates its use.

### 2.4 Overall marginal mean

When g(·) is the identity function, the overall marginal mean, $E(Y_{\mathrm{ij}})\equiv E(Y_{\mathrm{ij}}|\Xi_{\mathrm{ij}})$, is given by

$$E(Y_{\mathrm{ij}}|Y_{\mathrm{ij}}=0)\text{Pr}(Y_{\mathrm{ij}}=0)+E(Y_{\mathrm{ij}}|Y_{\mathrm{ij}}>0)\text{Pr}(Y_{\mathrm{ij}}>0)=E(Y_{\mathrm{ij}}|Y_{\mathrm{ij}}>0)\text{Pr}(Y_{\mathrm{ij}}>0)$$

where we have suppressed the dependence on the covariate vectors, $\Xi_{\mathrm{ij}}$, for convenience. Although a closed form for the overall marginal mean is not available, the analyst can easily numerically evaluate it (as is done in the subsequent Health Assessment Questionnaire (HAQ) analysis).

From Section 2.2, bounds on the overall marginal mean can be obtained as

$$\text{Pr}(Y_{\mathrm{ij}}>0)X_{\mathrm{ij}}^{*}\beta \leq E(Y_{\mathrm{ij}})\leq \text{Pr}(Y_{\mathrm{ij}}>0)X_{\mathrm{ij}}^{*}\beta +\frac{\sigma_{v}\rho }{\sqrt{2\pi }}$$

when ρ≥0, and

$$\text{Pr}(Y_{\mathrm{ij}}>0)X_{\mathrm{ij}}^{*}\beta \geq E(Y_{\mathrm{ij}})\geq \text{Pr}(Y_{\mathrm{ij}}>0)X_{\mathrm{ij}}^{*}\beta +\frac{\sigma_{v}\rho }{\sqrt{2\pi }}$$

when ρ≤0, where $\text{Pr}(Y_{\mathrm{ij}}>0)=(1+e-X_{\mathrm{ij}}\theta )-1$. Similar bounds can be derived for other common monotonic transformation functions for g(·). For example, bounds on $E(Y_{\mathrm{ij}}|\Xi_{\mathrm{ij}})$ when g(·) is logarithmic are shown in the Supplementary Material.

## 3 The HAQ data revisited

In this section, we revisit the HAQ data described in Su *et al.*^1^ The objective is to examine the association between alleles that code for human leukocyte antigen (HLA) proteins and disability level in a psoriatic arthritis (PsA) patient cohort. R code for this new analysis is located in the Supplementary Material.

Table 2 of Su *et al*.^1^ presented results from fitting the two-part mixed model to the data, where the third column shows the conditional covariate effects in the continuous part. As noted earlier, the corresponding marginal covariate effects are generally not equal to these conditional effects.

In this particular application, it is perhaps more natural to examine the association between the HLA alleles and the overall expected disability level of the patients over the study period, instead of the association when some disability is present. This is because disability, as measured by HAQ, for patients can vary over time and, for example, at one visit a patient can have mild disability, but at the next visit his/her situation may be improved resulting in a zero value of HAQ. We conjecture that it will often be felt to be clinically more informative to present the marginal covariate effects on the overall expected disability level together with the marginal covariate effects on the probability of having any level of disability.

For the HAQ example, we sample from the asymptotic distribution of the parameters based on the estimates in Table 2 of Su *et al*.^1^ and calculate the contrasts of overall expected HAQ with and without specific HLA alleles, controlling for other covariates. For presentation purposes, we fix the age at PsA diagnosis at 35 years and disease duration at 15 years, which correspond to zero values in standardized versions of the two variables. These contrasts represent the effects of HLA alleles on the overall expected disability level (controlling for other covariates) in the PsA patient cohort.

**Figure 1.**
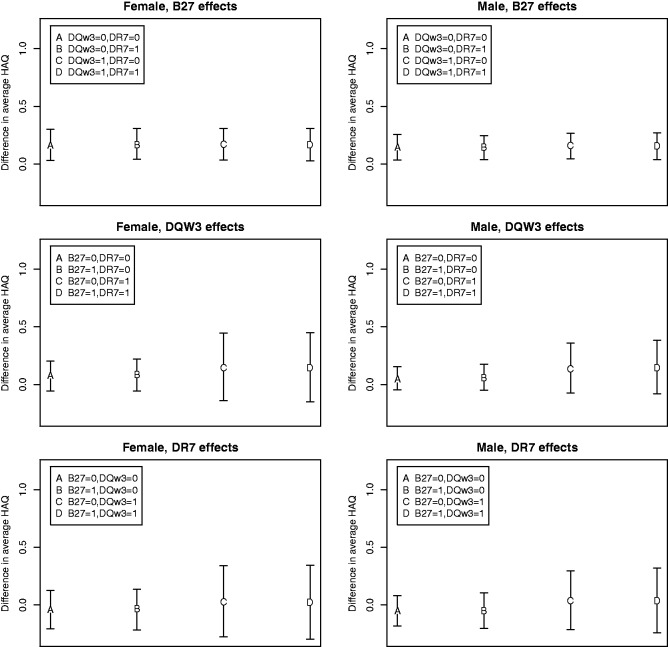
Contrasts (with 95% confidence intervals) of overall mean of HAQ for different combinations of the covariates (controlling for being 35 years old at PsA diagnosis and having a disease duration of 15 years). HAQ: Health Assessment Questionnaire.

The top panels of Figure 1 show the HLA-B27 effects given other alleles, sex, age at diagnosis and disease duration. Because the overall mean of HAQ is not directly parametrized in the fitted model, the corresponding covariate effects are not the same for all values of the other variables. However, the HLA-B27 effects are approximately the same across different combinations of other covariates, and the 95% confidence intervals do not include zero. This demonstrates a significant association between HLA-B27 and overall expected HAQ.

In Su *et al*.,^1^ we found a significant interaction between the effects of HLA-DQW3 and HLA-DR7 in the binary part of the two-part mixed model (p=0.035), while the same interaction was non-significant in the continuous part (p=0.85). The estimated marginal (log-odds ratio) effect of this interaction in the binary part was 0.8089 with 95% confidence interval [0.0565, 1.5613].

The middle and bottom panels of Figure 1 reflect the possible interaction between HLA-DQW3 and HLA-DR7 on the overall marginal mean of HAQ stratified by gender and absence/presence of the HLA-B27 allele. Age at PsA diagnosis is fixed at 35 years and disease duration at 15 years.

For illustrative purposes, considering the left middle (or bottom) panel of Figure 1 for females with the presence of HLA-B27, we estimate that the difference in the HLA-DQW3 (or, alternatively, HLA-DR7) effects on the overall marginal mean of HAQ between those with the presence of HLA-DR7 (or HLA-DQW3) allele and those with it absent (i.e. contrast D–B in figure) is 0.0564 with 95% confidence interval [–0.2062, 0.3232]. For females with HLA-B27 absent, the estimate of this difference in the HLA-DQW3 (or, alternatively, HLA-DR7) effects on the overall marginal mean of HAQ between those with and without the HLA-DR7 (or HLA-DQW3) allele (i.e. contrast C–A) is 0.0648 with 95% confidence interval [–0.1971, 0.3158]. These estimates of the HLA-DQW3 and HLA-DR7 interaction for females, with and without HLA-B27 present, are similar and both non-significant statistically. Conclusions based on these results are similar to those found for the continuous part in the two-part marginal model (data not shown).

## 4 Discussion

In this article, we have corrected the formulation for the continuous part of the two-part marginal model presented in Su *et al*.^1^ We show that the (marginal) mean of $g(Y_{\mathrm{ij}})|\Xi_{\mathrm{ij}},Y_{\mathrm{ij}}>0$ is not the fixed effects predictor, $X_{\mathrm{ij}}^{*}\beta$, as originally reported, but is non-linear in the covariates included in the binary part of the model. Thus, interpretation of the impact of a covariate on the marginal mean given being positive cannot be made from only considering the relevant component of β when the random effects are correlated and that covariate is also included in the binary part.

In some contexts, the logit may not be the preferred link function in the binary part. For example, in dilution and serological studies the cloglog link may be more appropriate. In psychometrics, the probit may be more convenient. For either of these alternatives, a two-part marginal formulation can be derived. For instance, if the logit link is replaced with the probit and *B_i_* is assumed $N(0,\sigma_{b}^{2})$ instead of from the bridge distribution when formulating the binary part of the two-part mixed model, then the link function of the marginal regression structure for the binary part, after integrating out *B_i_*, remains probit.^2^ Furthermore, in the binary part, the marginal covariate effects are proportional to their subject-specific conditional covariate effects, with constant of proportionality $1/\sqrt{1+\sigma_{b}^{2}}$. Under the same linear mixed effects structure considered earlier for the continuous part (i.e. *V_i_* normal and g(·) the identity function), a closed-form solution for the marginal mean of *Y_ij_*, given $Y_{\mathrm{ij}}>0$, (and therefore for the overall marginal mean) can be derived in terms of the standard normal density and cumulative distribution function (see Supplementary Material). Unfortunately, this closed-form solution is again non-linear in the covariates associated with the binary part, and therefore interpretation of marginal covariate effects on the continuous part (and on the overall marginal mean) will not generally be straightforward.

In conclusion, care should be taken when using two-part models for semi-continuous data that are longitudinal or otherwise clustered. Both the specification of random effects structures, as discussed in Su *et al*.,^14^ and the interpretation and calculation of marginal effects, as discussed in this paper, require careful attention to assumptions.

## Supplementary Material

Supplementary material
