# Supplementary material for: A corrected formulation for marginal inference derived from two-part mixed models for longitudinal semi-continuous data
Source: Stat Methods Med Res. 2013 Nov 6;25(5):2014–20. doi: 10.1177/0962280213509798 (PMC5051603; doi:10.1177/0962280213509798)
Supplement: Supplementary material [file Supplementary__Material.pdf]

Supplementary Material for "A corrected formulation for marginal inference derived from two-part mixed models for longitudinal semi-continuous data"

Brian D. M. Tom *, Li Su, and Vernon T. Farewell

Medical Research Council Biostatistics Unit, Robinson Way, Cambridge CB2 0SR, UK

Deriving the joint distribution of the random effects, V and B

Recall that in our proposed two-part mixed model formulation, V_i follows a mean zero normal distribution with variance σ_v^2 and B_i follows the (symmetric) mean zero bridge density of Wang and Louis[1]

$$f_{B_i}(b \mid \varphi) = \frac{1}{2\pi} \frac{\sin(\varphi\pi)}{\cosh(\varphi b) + \cos(\varphi\pi)} \quad (-\infty < b < \infty)$$

with unknown parameter φ $(0 < \varphi < 1)$ and variance $\sigma_b^2 = \pi^2(\phi^{-2} - 1)/3$.

The bivariate joint distribution of (B_i, V_i) is derived by considering a pair of normal random variables

$$\begin{bmatrix} U_i \\ V_i \end{bmatrix} \sim \mathrm{N}\left(\begin{bmatrix} 0 \\ 0 \end{bmatrix}, \begin{bmatrix} 1 & \rho\sigma_v \\ \rho\sigma_v & \sigma_v^2 \end{bmatrix} \right), \tag{0.1}$$

applying the probability integral transformation $B_i = F_{B_i}^{-1}\{\Phi(U_i)\}$ to obtain B_i[1,2], and then employing the change of variable method to arrive at the joint distribution. Here $\Phi(\cdot)$ is the cumulative distribution function of the standard Normal, ρ is the correlation between U_i and V_i and $F_{B_i}^{-1}(\cdot)$ is the inverse cumulative distribution function,

$$F_{B_i}^{-1}(x) = \frac{1}{\varphi} \log\left[\frac{\sin(\varphi\pi x)}{\sin\{\varphi\pi(1-x)\}} \right]$$

of the bridge density for $0 < x < 1$.

*Address for correspondence: Brian Tom, MRC Biostatistics Unit, Robinson Way, Cambridge CB2 0SR, UK. Email: brian.tom@mrc-bsu.cam.ac.uk; phone: 44-1223-330382

Expression and bounds for $E(g(Y) \mid Y > 0)$

As presented in the main text, the correct form of the marginal mean of $g(Y_{ij}) \mid \Xi_{ij}, Y_{ij} > 0$ is

$$E\{g(Y_{ij}) \mid \boldsymbol{\Xi}_{ij}, Y_{ij} > 0\} = \mathbf{X}^*_{ij}\boldsymbol{\beta} + E(V_i \mid \boldsymbol{\Xi}_{ij}, Y_{ij} > 0).$$

Evaluating for $E(V_i \mid \boldsymbol{\Xi}_{ij}, Y_{ij} > 0) \equiv E(V_i \mid \mathbf{X}_{ij}, Z_{ij} = 1)$ on the RHS of the above equation gives

$$\begin{aligned} E(V_i \mid \mathbf{X}_{ij}, Z_{ij} = 1) &= \int_{-\infty}^{\infty} v \int_{-\infty}^{\infty} \frac{f_{V_i|B_i}(v \mid b) f_{B_i}(b) \Pr(Z_{ij} = 1 \mid \mathbf{X}_{ij}, B_i = b)}{\Pr(Z_{ij} = 1 \mid \mathbf{X}_{ij})} db dv \\ &= -\sigma_v \rho (1 + e^{-\mathbf{X}_{ij}\boldsymbol{\theta}}) \int_{-\infty}^{\infty} \frac{\Phi^{-1}(F_{B_i}(b)) f_{B_i}(b)}{1 + e^{\mathbf{X}_{ij}\tilde{\boldsymbol{\theta}} + b}} db \\ &= -\sigma_v \rho (1 + e^{-\mathbf{X}_{ij}\boldsymbol{\theta}}) \int_{-\infty}^{\infty} \frac{u \phi(u) du}{1 + e^{\mathbf{X}_{ij}\tilde{\boldsymbol{\theta}} + F_{B_i}^{-1}(\Phi(u))}}, \end{aligned}$$

where the second line is obtained by interchanging the order of integration, noticing that $\Pr(Z_{ij} = 1 \mid X_{ij}) = (1 + e^{-\mathbf{X}_{ij}\boldsymbol{\theta}})^{-1}$ and $\int_{-\infty}^{\infty} \Phi^{-1}(F_{B_i}(b)) f_{B_i}(b) db = 0$, and then showing that the conditional distribution of $(V_i \mid B_i = b)$ is $N(\sigma_v \rho \Phi^{-1}(F_{B_i}(b)), \sigma_v^2(1 - \rho^2))$. The third line is obtained through use of the change of variable method with $u = \Phi^{-1}(F_{B_i}(b))$. It is therefore clear that $E(V_i \mid \boldsymbol{\Xi}_{ij}, Y_{ij} > 0)$, and thus $E\{g(Y_{ij}) \mid \boldsymbol{\Xi}_{ij}, Y_{ij} > 0\}$, are dependent on the impact of covariates, $\mathbf{X}_{ij}$, on the marginal and conditional probabilities of occurrence. $\phi(u)$ denotes the standard normal density function.

To obtain bounds on the marginal mean, $E\{g(Y_{ij}) \mid \boldsymbol{\Xi}_{ij}, Y_{ij} > 0\}$, we begin by considering

$$I = -\int_{-\infty}^{\infty} \frac{u \phi(u) du}{1 + e^{\mathbf{X}_{ij}\tilde{\boldsymbol{\theta}} + F_{B_i}^{-1}(\Phi(u))}}.$$

We next decompose I into two components

$$-\int_{-\infty}^{0} \frac{u \phi(u) du}{1 + e^{\mathbf{X}_{ij}\tilde{\boldsymbol{\theta}} + F_B^{-1}(\Phi(u))}} - \int_{0}^{\infty} \frac{u \phi(u) du}{1 + e^{\mathbf{X}_{ij}\tilde{\boldsymbol{\theta}} + F_B^{-1}(\Phi(u))}} \quad = \quad I_1 + I_2.$$

By noting the following relationship:

$$\int_0^{\infty} u \phi(u) du = 1/\sqrt{2\pi}; \tag{0.2}$$

and using the following constraints:

$$F_{B_i}^{-1}(\Phi(u)) \begin{cases} < 0 & \text{if } u < 0 \\ = 0 & \text{if } u = 0 \\ > 0 & \text{if } u > 0 \end{cases};$$

$0 \leq 1/(1+e^{\mathbf{X}_{ij}\tilde{\boldsymbol{\theta}}}) \leq 1/(1+e^{\mathbf{X}_{ij}\tilde{\boldsymbol{\theta}}+F_B^{-1}(\Phi(u))}) \leq 1$ for $u < 0$; and $1 \geq 1/(1+e^{\mathbf{X}_{ij}\tilde{\boldsymbol{\theta}}}) \geq 1/(1+e^{\mathbf{X}_{ij}\tilde{\boldsymbol{\theta}}+F_B^{-1}(\Phi(u))}) \geq 0$ for $u > 0$, we can show that

$$0 \leq \int_{-\infty}^{0} \frac{-u\phi(u)}{1+e^{\mathbf{X}_{ij}\tilde{\boldsymbol{\theta}}}} du \leq I_1 \leq \int_{-\infty}^{0} -u\phi(u) du \tag{0.3}$$

and

$$0 \geq I_2 \geq \int_{0}^{\infty} \frac{-u\phi(u)}{1+e^{\mathbf{X}_{ij}\tilde{\boldsymbol{\theta}}}} du. \tag{0.4}$$

Now using (0.3), (0.4) and (0.2), we see that

$$\begin{aligned} I_1 + I_2 &\geq \int_{-\infty}^{0} \frac{-u\phi(u)}{1+e^{\mathbf{X}_{ij}\tilde{\boldsymbol{\theta}}}} du + \int_{0}^{\infty} \frac{-u\phi(u)}{1+e^{\mathbf{X}_{ij}\tilde{\boldsymbol{\theta}}}} du \\ &\geq -(1+e^{\mathbf{X}_{ij}\tilde{\boldsymbol{\theta}}})^{-1} \int_{-\infty}^{\infty} u\phi(u) du \\ &\geq 0, \end{aligned}$$

and that

$$\begin{aligned} I_1 + I_2 &\leq \int_{-\infty}^{0} -u\phi(u) du + I_2 \\ &\leq -\int_{-\infty}^{0} u\phi(u) du - \int_{0}^{\infty} \frac{(1+e^{\mathbf{X}_{ij}\tilde{\boldsymbol{\theta}}+F_B^{-1}(\Phi(u))}) - e^{\mathbf{X}_{ij}\tilde{\boldsymbol{\theta}}+F_B^{-1}(\Phi(u))}}{1+e^{\mathbf{X}_{ij}\tilde{\boldsymbol{\theta}}+F_B^{-1}(\Phi(u))}} u\phi(u) du \\ &\leq \int_{0}^{\infty} \frac{e^{\mathbf{X}_{ij}\tilde{\boldsymbol{\theta}}+F_B^{-1}(\Phi(u))}}{1+e^{\mathbf{X}_{ij}\tilde{\boldsymbol{\theta}}+F_B^{-1}(\Phi(u))}} u\phi(u) du \\ &\leq \int_{0}^{\infty} u\phi(u) du \\ &\leq 1/\sqrt{2\pi}. \end{aligned}$$

Thus we arrive at $0 \leq I \leq 1/\sqrt{2\pi}$. Therefore for $\rho \geq 0$

$$\mathbf{X}_{ij}^{*}\boldsymbol{\beta} \leq E(g(Y_{ij}) \mid \boldsymbol{\Xi}_{ij}, Y_{ij} > 0) \leq \mathbf{X}_{ij}^{*}\boldsymbol{\beta} + \frac{\sigma_v \rho}{\sqrt{2\pi}}(1+e^{-\mathbf{X}_{ij}\boldsymbol{\theta}}),$$

and for $\rho \leq 0$,

$$\mathbf{X}_{ij}^{*}\boldsymbol{\beta} \geq E(g(Y_{ij}) \mid \boldsymbol{\Xi}_{ij}, Y_{ij} > 0) \geq \mathbf{X}_{ij}^{*}\boldsymbol{\beta} + \frac{\sigma_v \rho}{\sqrt{2\pi}}(1+e^{-\mathbf{X}_{ij}\boldsymbol{\theta}}).$$

Expression and bounds for $E(Y)$ when $g(Y) = \log(Y)$

When the transformation $g(\cdot)$ is logarithmic, then the overall marginal mean, $E(Y_{ij} \mid \boldsymbol{\Xi}_{ij})$, can be shown to be

$$E(Y_{ij} \mid \boldsymbol{\Xi}_{ij}) = e^{\mathbf{X}^*_{ij}\boldsymbol{\beta}+\frac{1}{2}(\sigma_e^2+\sigma_v^2(1-\rho^2))} \int_{-\infty}^{\infty} \frac{e^{\mathbf{X}_{ij}\tilde{\boldsymbol{\theta}}+F_{B_i}^{-1}(\Phi(u))}}{1+e^{\mathbf{X}_{ij}\tilde{\boldsymbol{\theta}}+F_{B_i}^{-1}(\Phi(u))}} e^{\sigma_v \rho u}\phi(u)du, \tag{0.5}$$

which can be evaluated numerically. Bounds on this overall marginal mean can be derived (for any correlation ρ) and are expressed as

$$e^{\mathbf{X}^*_{ij}\boldsymbol{\beta}+\frac{1}{2}(\sigma_e^2+\sigma_v^2(1-\rho^2))} \int_0^{\infty} e^{-2b} f_{B_i}(b)db \leq E(Y_{ij} \mid \Xi_{ij}) \leq e^{\mathbf{X}^*_{ij}\boldsymbol{\beta}+\frac{1}{2}(\sigma_e^2+\sigma_v^2)}. \tag{0.6}$$

Two-part mixed model under probit link and Gaussian random intercepts

If instead of using the logit link function in the binary part we use the probit link, and if we assume that (V_i, B_i) is bivariate normal with a zero mean vector and variance components, σ_v^2, σ_b^2, and correlation, ρ, then our new formulation of the two-part mixed model is $\Phi^{-1}\{\Pr(Z_{ij} = 1 \mid \boldsymbol{\Omega}_{ij})\} = \mathbf{X}_{ij}\tilde{\boldsymbol{\theta}} + B_i$ for the binary part, and $g(Y_{ij}) \mid \boldsymbol{\Omega}_{ij}, Y_{ij} > 0 = \mathbf{X}^*_{ij}\boldsymbol{\beta} + V_i + \epsilon_{ij}$ for the continuous part. The error, ϵ_{ij}, is again assumed to be $N(0, \sigma_e^2)$ and independent of the random effects.

Now

$$\begin{aligned}
\Pr(Z_{ij} = 1 \mid \boldsymbol{\Omega}_{ij}) &= \Pr(Z_{ij} = 1 \mid \mathbf{X}_{ij}) \\
&= \int_{-\infty}^{\infty} \Pr(Z_{ij} = 1 \mid \mathbf{X}_{ij}, B_i = b) f_{B_i}(b)db \\
&= \frac{1}{\sigma_b}\int_{-\infty}^{\infty} \Phi(X_{ij}\tilde{\boldsymbol{\theta}} + b)\phi(b/\sigma_b)db \\
&= \int_{-\infty}^{\infty} \Phi(X_{ij}\tilde{\boldsymbol{\theta}} + \sigma_b w)\phi(w)dw \\
&= \Phi(X_{ij}\boldsymbol{\theta}),
\end{aligned}$$

where $\boldsymbol{\theta} = \tilde{\boldsymbol{\theta}}/\sqrt{1+\sigma_b^2}$ and where the last line follows from applying 10,010.8 of Owen[3].

Furthermore,

$$E\{g(Y_{ij}) \mid \boldsymbol{\Xi}_{ij}, Y_{ij} > 0\} = \mathbf{X}^*_{ij}\boldsymbol{\beta} + E(V_i \mid \boldsymbol{\Xi}_{ij}, Y_{ij} > 0),$$

where now the expression for $E(V_i \mid \boldsymbol{\Xi}_{ij}, Y_{ij} > 0) \equiv E(V_i \mid \mathbf{X}_{ij}, Z_{ij} = 1)$ is given by

$$
\begin{aligned}
E(V_i \mid \mathbf{X}_{ij}, Z_{ij} = 1) &= \int_{-\infty}^{\infty} v \int_{-\infty}^{\infty} \frac{f_{V_i|B_i}(v \mid b) f_{B_i}(b) \Pr(Z_{ij} = 1 \mid X_{ij}, B_i = b)}{\Pr(Z_{ij} = 1 \mid X_{ij})} db dv \\
&= \frac{1}{\Pr(Z_{ij} = 1 \mid X_{ij})} \int_{-\infty}^{\infty} \Pr(Z_{ij} = 1 \mid X_{ij}, B_i = b) f_{B_i}(b) \int_{-\infty}^{\infty} v f_{V_i|B_i} dv db \\
&= \frac{\rho\sigma_v}{\sigma_b^2 \Phi(\mathbf{X}_{ij}\boldsymbol{\theta})} \int_{-\infty}^{\infty} b \Phi(\mathbf{X}_{ij}\tilde{\boldsymbol{\theta}} + b) \phi(b/\sigma_b) db \\
&= \frac{\rho\sigma_v}{\Phi(\mathbf{X}_{ij}\boldsymbol{\theta})} \int_{-\infty}^{\infty} w \Phi(\mathbf{X}_{ij}\tilde{\boldsymbol{\theta}} + \sigma_b w) \phi(w) dw \\
&= \frac{\rho\sigma_v\sigma_b}{\sqrt{1 + \sigma_b^2}} \frac{\phi(\mathbf{X}_{ij}\boldsymbol{\theta})}{\Phi(\mathbf{X}_{ij}\boldsymbol{\theta})},
\end{aligned}
$$

where the last line follows from applying 10,011.3 of Owen[3]. Thus

$$
E\{g(Y_{ij}) \mid \boldsymbol{\Xi}_{ij}, Y_{ij} > 0\} = \mathbf{X}_{ij}^{*}\boldsymbol{\beta} + \frac{\rho\sigma_v\sigma_b}{\sqrt{1 + \sigma_b^2}} \frac{\phi(\mathbf{X}_{ij}\boldsymbol{\theta})}{\Phi(\mathbf{X}_{ij}\boldsymbol{\theta})}.
$$

If $g(y) = y$, the identity transformation, then the above expression applies to the overall marginal mean of the response Y_{ij}.

R Program for the HAQ Analysis

The SAS code for the original two-part marginal analysis reported in Table 2 of Su *et al.*[4] can be found in the Supplementary Material of Su *et al.*[4]. The R code for the overall marginal mean analysis reported in this current paper is provided below.

```
#### calculate overall marginal mean for the two-part model fitted to the PsA data
#### in the SMMR paper


#### binary part logit(pr(y_ij>0))=logit(pr(z_ij=1)=X\theta+B_i
#### continuous part  yij|yij>0=Xstar\beta+V_i+e_ij
#### B_i=F^{-1}_B(\Phi(U_i)) follows the Bridge distribution with parameter \phi
#### (U_i, V_i)~N((0,0),(sigma_u, sigma01, sigma01, sigma_v)
####  sigma01=\rho*sqrt(sigma_u*sigma_v)


# function to compute overall marginal mean given covariate values and parameters
```

```
overallM<-function(X,theta,Xstar,beta,sigmav,rho,phi)
{
library(statmod)
etab=t(X)%*%theta # linear predictor in binary part
etac=t(Xstar)%*%beta # linear predictor in continuous part
out<-gauss.quad.prob(20,"normal", mu=0, sigma=1)
Mnumer<-(etac+sqrt(sigmav)*rho*out$nodes)*(sin(phi*pi*pnorm(out$nodes)))^(1/phi)
Mdenom<-(sin(phi*pi*(1-pnorm(out$nodes))))^(1/phi)+exp(etab)*
            (sin(phi*pi*pnorm(out$nodes)))^(1/phi)
M<-exp(etab)*sum(out$weights*Mnumer/Mdenom)
return(M)
}

#### point estimates and standard errors from Table 2 of Su et al. (2011)
theta<-c(1.2848 ,0.9736 ,-0.4620,-0.9786,1.6642 ,0.8206 ,0.3863 ,2.5067)
thetaSD<-c(0.3665,0.4535,0.4465,0.5869,0.7860,0.1838,0.1415,0.4093)
beta<-c(0.4563, 0.1652, 0.1075, -0.0158,0.0256, 0.1071, 0.0488, 0.3388)
betaSD<-c(0.0630,0.0756,0.0762,0.1023,0.1344,0.0289,0.0205,0.0630)
sigmav<-0.2851
sigmavSD<- 0.0261
rho<-0.9801
rhoSD<-0.0151
phi<-0.4861
phiSD<-0.0308

####  covariate values
####  Intercept, HLA-B27,HLA-DQw3,HLA-DR7,HLA-DQw3 and HLA-DR7 interaction,
####  Age at onset of (standarized, 35 years), PsA disease duration
```

```
####  (standardized, 15 years), Sex (Female)

B27<-c(0,1)
DQw3<-c(0,1)
DR7<-c(0,1)

#### function to compute overall marginal mean for females given other covariate
#### values, based on point estimates of parameters

getEstimate1<-function(theta,beta,sigmav,rho,phi)
{
   index<-matrix(c(1,1,1,2,1,1,1,1,2,2,1,2,1,2,1,2,2,1,1,2,2,2,2,2),nrow=8,ncol=3,byrow=T)
   estimate<-rep(0,8)
   for (i in 1:8)
{
    X<-c(1, B27[index[i,1]],DQw3[index[i,2]], DR7[index[i,3]],
            DQw3[index[i,2]]*DR7[index[i,3]], 0,0,1)
    estimate[i]<-overallM(X,theta,X,beta,sigmav,rho,phi)
   }
   return(estimate)
}

#### function to compute overall marginal mean for males given other covariate
#### values, based on point estimates of parameters

getEstimate2<-function(theta,beta,sigmav,rho,phi)
{
   index<-matrix(c(1,1,1,2,1,1,1,1,2,2,1,2,1,2,1,2,2,1,1,2,2,2,2,2),nrow=8,ncol=3,byrow=T)
```

```
   estimate<-rep(0,8)
   for (i in 1:8)
{
    X<-c(1, B27[index[i,1]],DQw3[index[i,2]],DR7[index[i,3]],
            DQw3[index[i,2]]*DR7[index[i,3]],0,0,0)
    estimate[i]<-overallM(X,theta,X,beta,sigmav,rho,phi)
   }
   return(estimate)
}

### compute point estimates of the overall marginal mean for females and males
point1<-getEstimate1(theta,beta,sigmav,rho,phi)
point2<-getEstimate2(theta,beta,sigmav,rho,phi)

### function to sample from asymptotic distributions of the parameters and
### obtain the samples for the overall marginal means (females)

getSD1<-function(n,theta,beta,sigmav,rho,phi, thetaSD,betaSD,sigmavSD,rhoSD,phiSD)
{
   i=1
   samples<-NULL
   while(i<=n)
{
newtheta<-theta
newbeta<-beta
for (j in 1:length(theta))
{
newtheta[j]<-rnorm(1,theta[j],thetaSD[j])
```

```
newbeta[j]<-rnorm(1,beta[j],betaSD[j])
}
newsigmav<-rnorm(1,sigmav,sigmavSD)
newrho<-rnorm(1,rho,rhoSD)
newphi<-rnorm(1,phi,phiSD)
point<-getEstimate1(newtheta,newbeta,newsigmav,newrho,newphi)
        samples<-rbind(samples,point)
        i=i+1
}
   return(samples)
}


### function to sample from asymptotic distributions of the parameters and
### obtain the samples for the overall marginal means (males)
getSD2<-function(n,theta,beta,sigmav,rho,phi, thetaSD,betaSD,sigmavSD,rhoSD,phiSD)
{
   i=1
   samples<-NULL
   while(i<=n)
{
newtheta<-theta
newbeta<-beta
for (j in 1:length(theta))
{
newtheta[j]<-rnorm(1,theta[j],thetaSD[j])
newbeta[j]<-rnorm(1,beta[j],betaSD[j])
}
newsigmav<-rnorm(1,sigmav,sigmavSD)
```

```
newrho<-rnorm(1,rho,rhoSD)
newphi<-rnorm(1,phi,phiSD)
point<-getEstimate2(newtheta,newbeta,newsigmav,newrho,newphi)
        samples<-rbind(samples,point)
        i=i+1
}
   return(samples)
}

##### obtain the samples for the overall marginal means (females, males)
repsample1<-getSD1(1000,theta,beta,sigmav,rho,phi, thetaSD,betaSD,sigmavSD,rhoSD,phiSD)
repsample2<-getSD2(1000,theta,beta,sigmav,rho,phi, thetaSD,betaSD,sigmavSD,rhoSD,phiSD)

#### calculate contrasts for B27, DQW3, DR7 and 95% confidence interval
 ind<-matrix(c(2,1,4,3,6,5,8,7,5,1,6,2,7,3,8,4,3,1,4,2,7,5,8,6),ncol=2,byrow=T)
 lim1<-NULL
 for(j in 1:12)
{
lim1<-rbind(lim1,c(point1[ind[j,1]]-point1[ind[j,2]],
            quantile(repsample1[,ind[j,1]]-repsample1[,ind[j,2]],probs=c(0.025,0.975))))
}
 lim2<-NULL
 for(j in 1:12)
{
lim2<-rbind(lim2,c(point2[ind[j,1]]-point2[ind[j,2]],
            quantile(repsample2[,ind[j,1]]-repsample2[,ind[j,2]],probs=c(0.025,0.975))))
}
```

```
####calculate interaction effect and 95% confidence interval
c((point1[7]-point1[3])-(point1[5]-point1[1]),
quantile(repsample1[,7]-repsample1[,3]-repsample1[,5]+repsample1[,1],probs=c(0.025,0.975)))

c((point1[8]-point1[4])-(point1[6]-point1[2]),
quantile(repsample1[,8]-repsample1[,4]-repsample1[,6]+repsample1[,2],probs=c(0.025,0.975)))

c((point1[7]-point1[5])-(point1[3]-point1[1]),
quantile(repsample1[,7]-repsample1[,5]-repsample1[,3]+repsample1[,1],probs=c(0.025,0.975)))

c((point1[8]-point1[6])-(point1[4]-point1[2]),
quantile(repsample1[,8]-repsample1[,6]-repsample1[,4]+repsample1[,2],probs= (0.025,0.975)))

###### plot the contrasts and 95% confidence intervals
postscript(file="~/overallmeanconstrast.ps",paper="special",height=10,width=8,title="",
            horizontal=FALSE,bg='white')

par(mfrow=c(3,2))

library(plotrix)

###B27 effects
par(xaxt='n')
plotCI(1:4,lim1[1:4,1],ui=lim1[1:4,3],li=lim1[1:4,2],pch=NA,gap=0.02,xlab='',
    ylab='Difference in average HAQ',ylim=c(-0.3,1.2),cex=1,main='Female, B27 effects')
text(1:4,lim1[1:4,1],LETTERS[1:4],cex=1)

legend(1,1.2,c('DQw3=0,DR7=0',
```

```
               'DQw3=0,DR7=1',
               'DQw3=1,DR7=0',
               'DQw3=1,DR7=1'),pch=LETTERS[1:4], pt.cex=1, ncol=1,bty='o',cex=1 )

par(xaxt='n')
plotCI(1:4,lim2[1:4,1],ui=lim2[1:4,3],li=lim2[1:4,2],pch=NA,gap=0.02,xlab='',
    ylab='Difference in average HAQ',ylim=c(-0.3,1.2),cex=1,main='Male, B27 effects')
text(1:4,lim2[1:4,1],LETTERS[1:4],cex=1)

legend(1,1.2,c('DQw3=0,DR7=0',
               'DQw3=0,DR7=1',
               'DQw3=1,DR7=0',
               'DQw3=1,DR7=1'),pch=LETTERS[1:4], pt.cex=1, ncol=1,bty='o',cex=1 )

#### DQW3 effects
par(xaxt='n')
plotCI(1:4,lim1[4+(1:4),1],ui=lim1[4+(1:4),3],li=lim1[4+(1:4),2],pch=NA,gap=0.02,xlab='',
    ylab='Difference in average HAQ',ylim=c(-0.3,1.2),cex=1,main='Female, DQW3 effects')
text(1:4,lim1[4+(1:4),1],LETTERS[1:4],cex=1)

legend(1,1.2,c('B27=0,DR7=0',
               'B27=1,DR7=0',
               'B27=0,DR7=1',
               'B27=1,DR7=1'),pch=LETTERS[1:4], pt.cex=1, ncol=1,bty='o',cex=1 )

par(xaxt='n')
plotCI(1:4,lim2[4+(1:4),1],ui=lim2[4+(1:4),3],li=lim2[4+(1:4),2],pch=NA,gap=0.02,xlab='',
    ylab='Difference in average HAQ',ylim=c(-0.3,1.2),cex=1,main='Male, DQW3 effects')
```

```
text(1:4,lim2[4+(1:4),1],LETTERS[1:4],cex=1)

legend(1,1.2,c('B27=0,DR7=0',
               'B27=1,DR7=0',
               'B27=0,DR7=1',
               'B27=1,DR7=1'),pch=LETTERS[1:4], pt.cex=1, ncol=1,bty='o',cex=1 )

#### DR7 effects
par(xaxt='n')
plotCI(1:4,lim1[8+(1:4),1],ui=lim1[8+(1:4),3],li=lim1[8+(1:4),2],pch=NA,gap=0.02,xlab='',
    ylab='Difference in average HAQ',ylim=c(-0.3,1.2),cex=1,main='Female, DR7 effects')
text(1:4,lim1[8+(1:4),1],LETTERS[1:4],cex=1)

legend(1,1.2,c('B27=0,DQw3=0',
               'B27=1,DQw3=0',
               'B27=0,DQw3=1',
               'B27=1,DQw3=1'),pch=LETTERS[1:4], pt.cex=1, ncol=1,bty='o',cex=1 )

par(xaxt='n')
plotCI(1:4,lim2[8+(1:4),1],ui=lim2[8+(1:4),3],li=lim2[8+(1:4),2],pch=NA,gap=0.02,xlab='',
    ylab='Difference in average HAQ',ylim=c(-0.3,1.2),cex=1,main='Male, DR7 effects')
text(1:4,lim2[8+(1:4),1],LETTERS[1:4],cex=1)

legend(1,1.2,c('B27=0,DQw3=0',
               'B27=1,DQw3=0',
               'B27=0,DQw3=1',
               'B27=1,DQw3=1'),pch=LETTERS[1:4], pt.cex=1, ncol=1,bty='o',cex=1 )
dev.off()
```

References

1 Wang Z, Louis T. Matching conditional and marginal shapes in binary mixed-effects models using a bridge distribution function. *Biometrika* 2003; 90: 765–775.

2 Lin L, Bandyopadhyay D, Lipsitz SR, Sinha D. Association models for clustered data with binary and continuous responses. *Biometrics* 2010; 66: 287–293.

3 Owen DB. A table of normal integrals. *Communications in Statistics - Simulation and Computation* 1980; 9: 389–419.

4 Su L, Tom B, Farewell V. A likelihood-based two-part marginal model for longitudinal semicontinuous data. *Statistical Methods in Medical Research* 2013; DOI: 10.1177/0962280211414620.
